# Fatigue‐Resistant Mechanoresponsive Color‐Changing Hydrogels for Vision‐Based Tactile Robots

**DOI:** 10.1002/adma.202407925

**Published:** 2024-09-27

**Authors:** Jiabin Liu, Wei Li, Yu She, Sean Blanchard, Shaoting Lin

**Affiliations:** ^1^ Department of Mechanical Engineering Michigan State University East Lansing MI 48824 USA; ^2^ Department of Civil Engineering Stony Brook University Stony Brook NY 11794 USA; ^3^ Department of Industrial Engineering Purdue University West Lafayette IN 47907 USA

**Keywords:** fatigue resistant, photoelasticity, soft materials, tactile sensor

## Abstract

Mechanoresponsive color‐changing materials that can reversibly and resiliently change color in response to mechanical deformation are highly desirable for diverse modern technologies in optics, sensors, and robots; however, such materials are rarely achieved. Here, a fatigue‐resistant mechanoresponsive color‐changing hydrogel (FMCH) is reported that exhibits reversible, resilient, and predictable color changes under mechanical stress. At its undeformed state, the FMCH remains dark under a circular polariscope; upon uniaxial stretching of up to six times its initial length, it gradually shifts its color from black, to gray, yellow, and purple. Unlike traditional mechanoresponsive color‐changing materials, FMCH maintains its performance across various strain rates for up to 10 000 cycles. Moreover, FMCH demonstrates superior mechanical properties with fracture toughness of 3000 J m^−2^, stretchability of 6, and fatigue threshold up to 400 J m^−2^. These exceptional mechanical and optical features are attributed to FMCH's substantial molecular entanglements and desirable hygroscopic salts, which synergistically enhance its mechanical toughness while preserving its color‐changing performance. One application of this FMCH as a tactile sensoris then demonstrated for vision‐based tactile robots, enabling them to discern material stiffness, object shape, spatial location, and applied pressure by translating stress distribution on the contact surface into discernible images.

## Graphical abstract

Mechanoresponsive color‐changing materials that can reversibly and resiliently change color in response to stress are highly desirable for diverse technologies in optics, sensors, and robots; however, such materials are rarely achieved. This work reports a fatigue‐resistant mechanoresponsive color‐changing hydrogel that exhibits reversible, resilient, and predictable color changes under mechanical stress, for tactile robots by translating tactile sensations into visual images.

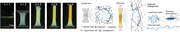

## 1 Introduction

High‐performing mechanoresponsive color‐changing materials that can reversibly and resiliently change color in response to mechanical deformation^[^ ^1^ ^]^ are highly desirable for diverse modern technologies such as display textiles for optical sensing,^[^ ^2^ ^]^ optical fibers for robotic proprioception,^[^ ^3^ ^]^ and molecular probes for damage visualization.^[^ ^4^ ^]^ Specifically, for modern tactile robots, these high‐performing mechanoresponsive color‐changing materials play an important role in translating tactile sensations into discernible images.^[^ ^5^ ^]^

Existing efforts to develop mechanoresponsive color‐changing materials typically rely on chemical synthesis of mechanophores,^[^ ^6^ ^]^ surface engineering of micro/nano‐structures,^[^ ^7^ ^]^ and molecular incorporation of electro/thermo/chemo‐chromic substances.^[^ ^8^ ^]^ Despite their progress, these efforts face several significant challenges that prevent their practical deployment, including poor cyclic performance, rate and angle dependence, slow responsiveness, and complex integration. Specifically, mechanoresponsive color‐changing materials based on mechanophore synthesis^[^ ^6^, ^9^ ^]^ often deteriorate under cyclic loading because their color transition relies predominantly on the scission of polymer chains. Although some mechanophores can demonstrate reversible color changes under moderate deformations through reversible sliding rings,^[^ ^10^ ^]^ they still lose their color‐changing ability after 10 to 50 cycles of loading under large deformations that inevitably cause chain scissions in the bulk material (Figure S1a, Supporting Information). In contrast, mechanoresponsive color‐changing materials based on structural engineering do not generally require chain scission or network transformation, yet they often exhibit angle‐dependent color changes due to their ordered structures, posing inherent challenges for consistent, reproducible, and predictable color‐changing responses.^[^ ^7^ ^]^ Furthermore, while incorporating electro/thermo/chemo‐chromic substances introduces additional design spaces for developing mechanoresponsive color‐changing materials, it also brings inherent limitations such as delayed response times due to the time required for field transmission^[^ ^8a^ ^]^ and added manufacturing complexity associated with their integration.^[^ ^11^ ^]^ Moreover, nearly all polymer‐based mechanoresponsive color‐changing materials experience significant rate dependence due to their viscoelasticity,^[^ ^12^ ^]^ thereby leading to rate‐dependent color‐changing performance. Therefore, there remains a critical need to innovate mechanoresponsive color‐changing materials that can address these limitations, offering rapid, reversible, and resilient color‐changing performance suitable for practical applications.

One attractive approach for developing ideal mechanoresponsive color‐changing materials is photoelasticity. Photoelasticity, a stress‐induced birefringence effect of transparent materials discovered in 1815,^[^ ^13^ ^]^ is currently receiving a resurgence of interest in soft materials^[^ ^14^ ^]^ due to its ability to probe large deformation of soft materials,^[^ ^15^ ^]^ quantify effective stress in porous media,^[^ ^16^ ^]^ and examine force chains in 3D granular media^[^ ^15b^ ^]^ . Distinctly different from mechanophore synthesis where color changes result from the scission of these chains,^[^ ^17^ ^]^ the color change in photoelasticity soft materials stems from the nonlinear elasticity of polymer chains; also intrinsically different from structural engineering, the color change in photoelasticity soft materials remains consistent across different viewing angles. Despite these significant advantages, existing photoelastic soft materials^[^ ^18^ ^]^ still suffer from fatigue fracture under multiple cycles of mechanical load,^[^ ^18^, ^19^ ^]^ a prevalent issue across many soft tough materials. As illustrated in Figure S1b (Supporting Information), repeated cycling causes molecular damage in existing photoelastic soft materials, altering chain alignment and leading to variable color‐changing responses over time. While photoelastic soft materials show great promise as mechanoresponsive color‐changing materials, further research is crucial to address these challenges, particularly for applications requiring durable and dynamic color‐changing capabilities.^[^ ^20^ ^]^

In this work, we report a fatigue‐resistant mechanoresponsive color‐changing hydrogel (FMCH) that consists of densely entangled polymer networks^[^ ^21^ ^]^ and optimally incorporated hygroscopic salts, exhibiting reversible, resilient, and predictable color changes under circular polariscope when subjected to mechanical stress. Notably, the FMCH undergoes a color shift from black to gray, yellow, and purple when uniaxially stretched to six times its initial length. Such mechanoresponsive color‐changing performance can be reliably maintained across a wide range of strain rates, from 0.02 to 0.35 s⁻¹ and remains functional for up to 10 000 cycles without loss of performance. Additionally, our FMCH demonstrates superior mechanical properties with fracture toughness of 3000 J m^−2^, stretchability of 6, and fatigue‐threshold up to 400 J m^−2^. These exceptional mechanical and optical properties are attributed to the FMCH's substantial molecular entanglements and optimally incorporated hygroscopic salts, which synergistically enhance its mechanical toughness while preserving its mechanical and photoelastic properties. Furthermore, we develop a low‐cost, long‐lasting, vision‐based tactile sensor that leverages our FMCH and reflective photoelasticimetry, capable of translating tactile sensations into visible color changes. We demonstrate our FMCH to enable the tactile robot for discerning material modulus, object shape, spatial positioning, and applied pressure through the direct visualization of stress distribution, further validated by finite element simulations. This work has the potential to fill the fundamental gap between robotic tactile perception and human haptic sensing.

## 2 Results and Discussion

### 2.1 Design of FMCH

**Figure 1:**
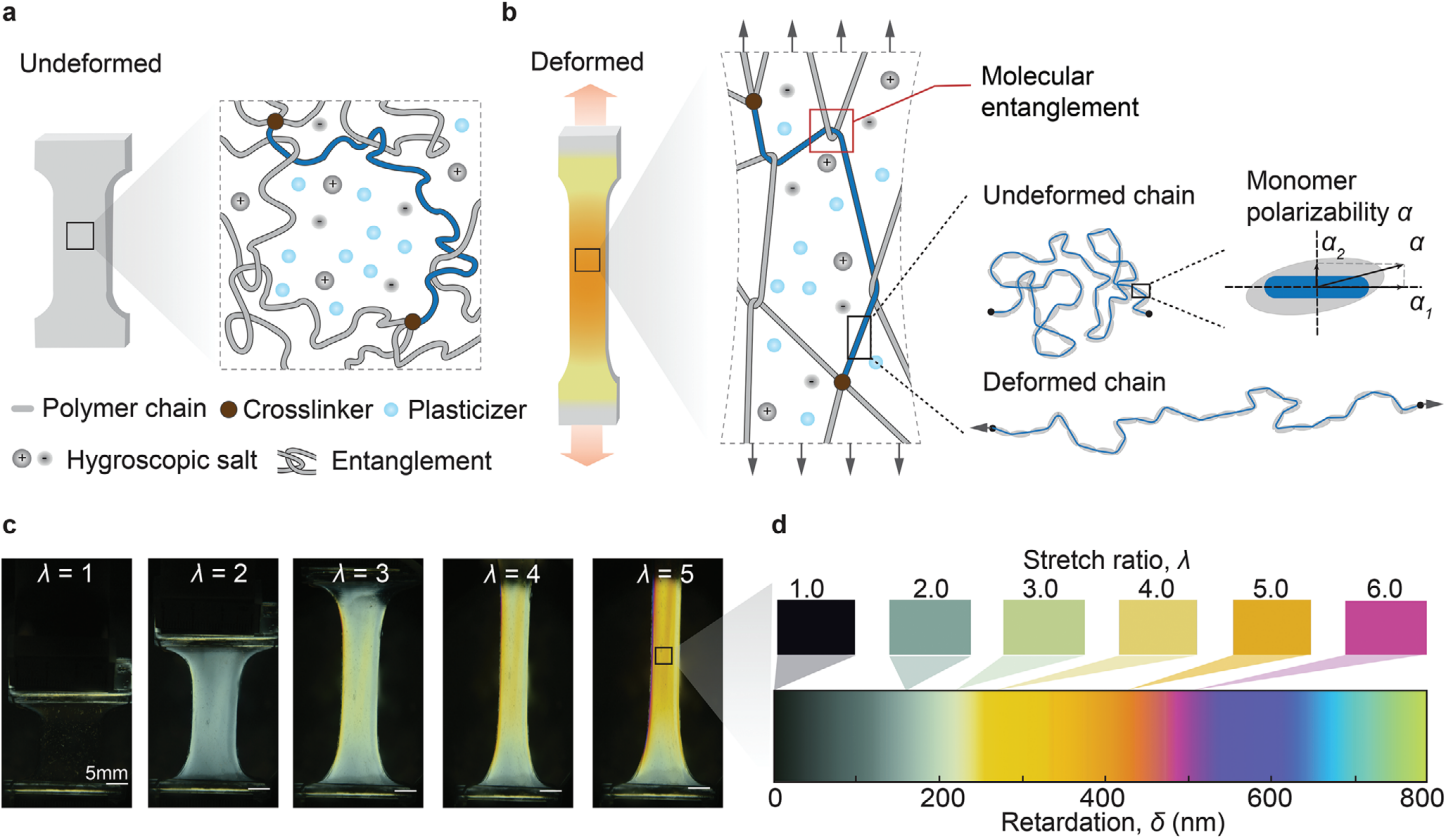
Molecular design and photoelasticity of FMCHs. a) The FMCH is composed of long polymer chains, crosslinkers, entanglements, hygroscopic salts, and plasticizers. b) When the FMCH is subjected to a tensile stretch, the molecular entanglements act as slip links, allowing the polymer chains to collectively bear the force. Concurrently, the alignment and orientation of polymer chains lead to a polarizability difference between the two principal stress directions (α_1_ parallel to the stretch direction and α_2_ perpendicular to the stretch direction), resulting in a stress‐dependent color change observable under a circular polariscope. c) The photoelastic images of the FMCH subjected to various stretch levels under a circular polariscope. d) Experimental photoelastic color and its corresponding retardation δ of the FMCH subjected to various stretch ratios λ.

The FMCH is composed of long polymer chains, crosslinkers, entanglements, hygroscopic salts, and plasticizers as illustrated in **Figure** **1**a. Each of these long polymer chains consists of a sequence of repeating monomer units, and each monomer possesses a dipole characterized by polarizability α (Figure 1b).^[^ ^22^ ^]^ At its undeformed state, the monomer dipoles within the polymer chain are randomly oriented, resulting in no directional difference in polarizability throughout the FMCH. As the FMCH is stretched, the dipoles of the monomers align and orient along the stretch direction. This alignment and orientation results in a polarizability difference between the two principal stress directions (α_1_ parallel to the stretch direction, α_2_ perpendicular to the stretch direction), which in turn causes a difference in refractive index (known as birefringence Δ*n*).^[^ ^23^ ^]^ When the light passes through the stretched FMCH, its electromagnetic wave components split along these two principal stress directions, resulting in an observable stress‐dependent color change under a circular polariscope (Figure 1c), namely photoelasticity.

To further engineer the FMCH with fatigue‐resistant photoelasticity, we design its long polymer chains to be densely intertwined, featuring substantial molecular entanglements supplemented by a few crosslinkers.^[^ ^21^, ^24^ ^]^ As shown in Figure 1b, when subjected to uniaxial tensile stress, these long polymer chains stretch and slide against each other, effectively distributing the stress without undergoing breakage. Additionally, we incorporate hygroscopic salts into the FMCH to prevent dehydration,^[^ ^25^ ^]^ ensuring its stable performance in ambient environments. The presence of hygroscopic salts retains plasticizers (i.e., water molecules), thereby reducing the inter‐chain friction and enabling the long polymer chains to slide smoothly against each other under mechanical stress. The reduced friction prevents chain breakage during extended cyclic loading and ensures a consistent optical response across different loading rates. The synergistic effects of the densely entangled polymer networks and optimally incorporated hygroscopic salts allow the FMCH to exhibit reversible, resilient, and predictable color changes when subjected to mechanical stress in ambient environment.

### 2.2 Material Synthesis

**Figure 2:**
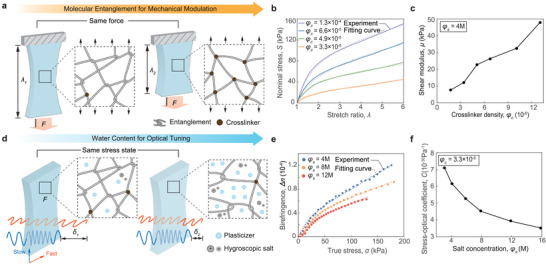
Mechanical‐optical characterizations of FMCHs. a) Schematic illustration of tuning molecular entanglement for mechanical modulation. The degree of molecular entanglements is tuned by altering the amount of crosslinkers. b) The measured nominal stress *S* versus stretch ratio λ of FMCHs with various crosslinker density φ_c_, fitted by one‐term Ogden hyperelastic model. c) The identified shear modulus μ versus crosslinker density φ_c_ for the FMCH with hygroscopic salt concentrations φ_s_ =  4 M. d) Schematic illustration of changing water content for optical tuning. The water content is regulated by adjusting the amount of hygroscopic salt. e) The measured birefringence versus true stress σ of various hygroscopic salt concentrations φ_s_, fitted by nonlinear photoelastic model. f) The extracted stress‐optical coefficient *C* versus salt concentration increases φ_s_ for the FMCH with crosslinker density φ_c_ =  3.3 × 10^−5^.

We synthesize a series of FMCHs with various crosslinker densities φ_c_ (**Figure** **2**a) and hygroscopic salt concentrations φ_*s*_ (Figure 2d) to experimentally investigate the impacts of molecular entanglements and plasticizer content (i.e., water content, the mass ratio of the water to the entire hydrogel at its as‐prepared state). The crosslinker density φ_c_, defined as the molar ratio between crosslinker and monomer, ranges from 1.6 × 10^−5^ to 1.6 × 10^−4^. To achieve dense entanglements, we prepare the precursor solutions using high concentrations of monomers, with a water‐to‐monomer molar ratio of approximately 4. This high concentration of monomers forms dense polymer chains, resulting in extensive entanglements.^[^ ^21b^ ^]^ For as‐synthesized hydrogels with the same polymer content (i.e., the mass ratio of the dry polymer to the entire hydrogel at its as‐prepared state), controlled increase of crosslinker density leads to a reduction in the average chain length, effectively restricting entanglements in the hydrogel.^[^ ^24^ ^]^ To efficiently incorporate hygroscopic salts into as‐synthesized hydrogel, we submerge the as‐synthesized hydrogels in deionized water until fully swollen (Figure S3, Supporting Information). Subsequently, the fully swollen hydrogels are submerged into LiCl solutions with varying concentration φ_*s*_, ranging from 2 to 16 mol L^−1^, to incorporate the hygroscopic salts. After the hygroscopic salts uniformly diffuse throughout the hydrogels, we place the hydrogels in the air until the water content in the hydrogels reaches its equilibrium state in ambient environment (Figure S4, Supporting Information). When immersed in a high concentration of salt solution, the hydrogel retains more hygroscopic salts, thereby enhancing its ability to retain water in the ambient environment. As shown in Figure S5b (Supporting Information), the water content, which is defined as the ratio between mass of water and mass of water and polymer, increases from 72% (2 mol L^−1^ LiCl) to 89% (16 mol L^−1^ LiCl) in an ambient environment with 50% humidity.

### 2.3 Mechanical and Optical Characterizations

We first conduct uniaxial tensile tests to measure the relationship between nominal stress and stretch ratios of FMCHs with controlled crosslinker densities φ_c_ and hygroscopic salt concentrations φ_s_ (Figure 2b). The shear modulus μ is determined by fitting the nominal stress–stretch curve to the one‐term Ogden hyperelastic model^[^ ^26^ ^]^ (Figure S6a, Supporting Information). As shown in Figure 2c, the shear modulus μ increases with the crosslinker density φ_c_, ranging from 8 to 50 kPa. This increase is attributed to the presence of the crosslinkers that constrain the mobility of polymer chains, thereby reducing the hydrogel's ability to deform and consequently resulting in increased modulus. Conversely, an increase in salt concentration φ_*s*_ leads to a slight decrease in shear modulus from 15 to 5 kPa (Figure S6b, Supporting Information). This reduction is due to the ability of hygroscopic salts to retain water molecules, which effectively dilutes the concentration of crosslinkers, resulting in a decreased modulus.

We further develop a photoelasticimetry experimental setup to investigate the impact of molecular entanglements and plasticizer contents on the photoelastic behavior of FMCHs. As shown in Figure S2 (Supporting Information), the photoelasticimetry experimental setup is composed of a light source, two linear polarizers, two quarter wave plates, a universal testing machine with photoelastic samples, and a camera (More details on the design of the photoelasticimetry experimental setup is provided in Supporting Information). We use the setup to measure the color in the middle region of the dog‐bone‐shaped specimens at different stretch ratios (Figure 1d). By comparing the measured color with the Michel‐Levy Birefringence Chart (Figure S7b, Supporting Information), we can identify the retardation δ, following *I*  =  *I*_0_sin ^2^(πδ/λ) where *I*_0_ is the input intensity, *I* is the measured intensity, and λ is the wavelength of the input light. Physically, the retardation defines the path difference of a light passing through the specimen between the light oscillating along fast axis (*h*ν_∥_, perpendicular to stretch direction) and the light oscillating along slow axis (*h*ν_⊥_, parallel to stretch direction). By dividing the retardation δ by the thickness of the specimen *d*, we can extract the birefringence of the material Δ*n*  =  δ/*d*. As shown in Figure 2e, different from traditional photoelastic materials, the birefringence Δ*n* of the FMCHs exhibits an intriguing nonlinear relationship with true stress σ. For highly deformable soft materials, the polymer chains will reach their limiting stretch, causing a non‐Gaussian chain effect and leading to a nonlinear stress‐stretch relationship. With the inclusion of non‐Gaussian chain effect, birefringence becomes dependent not only on the stretch ratio but also on the chain length *n* (i.e., the number of monomers in an individual polymer chain), leading to a nonlinear relationship between birefringence Δ*n* and true stress σ.^[^ ^27^ ^]^ Our experimental results further indicate the birefringence of FMCHs slightly increases as the material's hygroscopic salt concentration φ_*s*_ decreases (Figure 2e), which is attributed to the higher polymer content in FMCHs with lower hygroscopic salts concentrations that primarily influences the photoelastic effect of the material. We further measure the birefringence Δ*n* versus the stretch ratio λ of the FMCHs with various crosslinker densities φ_c_. As shown in Figure S8b (Supporting Information), while the measured birefringence Δ*n* shows little dependence on the crosslinker density φ_*c*_ at small deformation, it increases significantly with the crosslinker density φ_c_ at large deformation. This is possibly due to the pronounced alignment of polymer chains in FMCHs with high crosslinker density, where the chains approach their locking stretch at these large deformations.

To further quantify the nonlinear stress‐birefringence relationship of FMCHs, we adopt a nonlinear photoelastic model,^[^ ^28^ ^]^ writing as

(1) $$\frac{\Delta n}{\sigma_{1}-\sigma_{2}}=\;\frac{5C}{\mathrm{kT}}\frac{\sqrt{n}L^{-1}\left(\Lambda /\sqrt{n}\right)-3\Lambda }{\Lambda L^{-1}{\left(\Lambda /\sqrt{n}\right)}^{2}}$$

where *C* is the stress‐optical coefficient, which quantifies the relationship between the applied stress and the resulting birefringence in a material, *L* ^−1^(·) is the inverse Langevin function, and $\Lambda =\sqrt{(\lambda^{2}+2/\lambda )/3}$ is the stretch of individual polymer chain with λ being the uniaxial tensile stretch. As shown in Figure 2f, the stress‐optical coefficient *C* decreases significantly as the hygroscopic salt concentration φ_*s*_ increases, because the photoelastic effect of the FMCHs is mainly dominated by FMCHs’ polymer contents that decrease as the salt concentration increases. Specifically, according to the Treloar's theory,^[^ ^29^ ^]^ the stress‐optical coefficient *C* of the FMCHs is dominated by the contrast between the reflection index of the polymer (*n*_polymer_ = 1.45)^[^ ^30^ ^]^ and the reflection index of the solvent (*n*_water_ = 1.33),^[^ ^31^ ^]^ which decreases as the water content increases. We also measure the stress‐optical coefficient *C* as a function of the crosslinker density φ_*c*_, which exhibits little dependence (Figure S8, Supporting Information).

### 2.4 Fracture and Fatigue Characterizations

**Figure 3:**
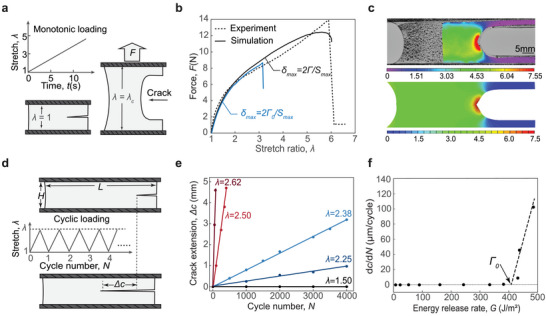
Fracture and fatigue characterizations of the FMCHs. a) Schematic illustration of pure‐shear tensile test to measure fracture toughness under a single cycle of load. b) Comparison of force versus stretch ratio curves between experiment and simulation for the notched FMCH under pure‐shear tensile test. The simulation with δ_max_ =  2Γ/*S*_max_ matches well with the experimental result, while the simulation with δ_max_ =  2Γ_0_/*S*_max_ is lower than the experimental result. c) Comparison of strain fields for the notched FMCH at the stretch ratio λ  =  3 between experiment using digital image correlation (DIC) and simulation using finite element modeling. d) Schematic illustration of pure‐shear tensile test to measure fatigue threshold under multiple cycles of load. e) The measured crack extension Δ*c* versus cyclic number *N* for the FMCH under different stretch ratios λ. f) The extracted crack extension per cycle d*c*/d*N* versus energy release rate *G*, which is correlated with the stretch ratio via $G\;=\;H\int_{1}^{\lambda }Sd\lambda$. The intersection of the curve at the abscissa identifies the fatigue threshold about 400 J m^−2^.

We carry out mechanical tests to measure fracture toughness Γ and fatigue threshold Γ_0_ of an optimal FMCH (φ_c_ = 3.3 × 10^−5^, φ_*s*_ = 4 M) to evaluate its mechanical robustness. Physically, the fracture toughness characterizes the material's ability to resist crack propagation under a single cycle of load, and the fatigue threshold characterizes the material's ability to resist crack propagation under multiple cycles of load.^[^ ^19^ ^]^ To measure the fracture toughness, we first carry out pure‐shear tensile tests to measure the nominal stress versus stretch ratio of an unnotched sample (Figure S9c, Supporting Information). Subsequently, we carry out pure‐shear tensile tests on a notched sample with the same dimensions as the unnotched sample, as depicted in **Figure** **3**a, undergoes the pure shear monotonic loading test to measure the critical stretch λ_*c*_, which is defined as the ratio between the sample length before crack propagation and its initial length. Given the measured critical stretch λ_c_, we further calculate the measured fracture toughness of the optimal FMCH about 3000 J m^−2^, following $\Gamma =H\int_{1}^{\lambda c}Sd\lambda$, where *H* is the initial length of the sample, *S* and λ are the nominal stress and the stretch ratio applied on the sample. To measure the FMCH's fatigue threshold Γ_0_, we carry out cyclic loading pure‐shear tensile loading on a notched FMCH at various stretch ratios λ (Figure 3d). Figure 3e plots the measured crack extension Δ*c* as a function of cycle number *N*, the slope of which measures the crack extension rate d*c*/d*N* increasing with the stretch ratio λ. Furthermore, we plot the crack extension rate d*c*/d*N* as a function of its corresponding energy release rate *G* (Figure 3f), which is correlated with the stretch ratio via $G=H\int_{1}^{\lambda }Sd\lambda$. The fatigue threshold of the optimal FMCH is identified approximately 400 J m^−^^2^ from the intersection of Δ*c*/Δ*N* versus *G* at the *G* axis.

To investigate the toughening mechanism of the optimal FMCH, we use the coupled cohesive and nonlinear elastic model implemented in finite element software Abaqus to capture the crack propagation in the notched FMCH.^[^ ^32^ ^]^ The material constant μ and α of the nonlinear elastic model are extracted by fitting the measured stress‐stretch curve from pure shear tensile tests into the one‐term Ogden hyperelastic model (Figure S9c, Supporting Information). We adopt the bilinear cohesive zone model to define the failure of material, characterized by the maximum stress *S*_max_ and the maximum separation δ_max_. The maximum stress *S*_max_ is taken as the measured nominal stress at failure under pure shear test, and the maximum separation δ_max_ is identified using δ_max_ =  2Γ/*S*_max_ with Γ being the fracture toughness of the FMCH. Traditionally, the maximum nominal separation δ_max_ should be identified using δ_max_ =  2Γ_0_/*S*_max_.^[^ ^32^ ^]^ As shown in Figure 3b, the simulation with δ_max_ =  2Γ/*S*_max_ matches well with the experimental result, while the simulation with δ_max_ =  2Γ_0_/*S*_max_ is significantly lower than the experimental result. This indicates the toughening of the FMCH does not follow the traditional high hysteresis toughening mechanism adopted in most soft tough materials.^[^ ^33^ ^]^ Conversely, the toughening of the FMCH relies on a low hysteresis toughening mechanism recently reported in highly entangled soft materials.^[^ ^21^, ^24^ ^]^ As shown in Figure 3c and Figure S9 (Supporting Information), we further carry out the digital image correlation (DIC) method to measure the strain field around crack tip in the notched FMCH, which matches well with our prediction in simulation with δ_max_ =  2Γ/*S*_max_.

### 2.5 Long‐Term Dynamic and Static Photoelastic Characterizations

We further conduct both dynamic and static photoelastic tests to assess the long‐term mechanoresponsive color‐changing performance of our FMCH. For comparison, we prepare three controlled samples: 1) Control sample 1, composed of the same polymer network as our FMCH but containing no hygroscopic salts, represents a typical entangled hydrogel, 2) Control sample 2, made of an interpenetrating polymer network, represents a typical tough hydrogel, and 3) Control sample 3, composed of the same polymer network as our FMCH but including glycerol as the plasticizer, represents a typical viscoelastic gel.

**Figure 4:**
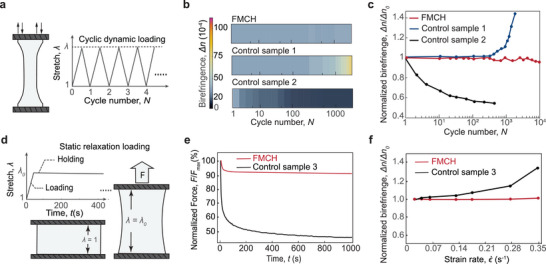
Long‐term dynamic and static photoelastic characterizations of the FMCHs. a) Schematic illustration of dynamic cyclic test. b) Comparisons of the photoelastic color and c) corresponding birefringence Δ*n* as a function of cycle number *N* among our FMCH and the other two control samples. d) Schematic illustration of static relaxation test. e) Comparison of the measured normalized force *F*/*F*_max_ as a function of time *t* between our FMCH and the control sample 3. f) Comparison of the measured normalized birefringence Δ*n*/Δ*n*_0_ as a function of strain rate $\dot{\varepsilon }$ at the stretch ratio of λ  =  3 between our FMCH and the control hydrogel 3, where Δ*n*_0_ denotes the birefringence at $\dot{\varepsilon }=0.02$ s^−1^.

We first perform dynamic cyclic photoelastic characterizations to evaluate our FMCH's fatigue‐resistant mechanoresponsive color‐changing performance and compare its performance with that of conventional tough hydrogels (Figure S10, Supporting Information). As shown in **Figure** **4**a, the FMCH is subjected to cyclic loading on a universal mechanical tester, recording its birefringence Δ*n* as a function of cycle number *N*. As shown in Figure 4b and the corresponding birefringence value in Figure 4c, the birefringence of our FMCH remains nearly unchanged after up to 10 000 cycles, demonstrating its fatigue‐resistant, mechanoresponsive color‐changing performance under long‐term dynamic loading. Due to the synergy of molecular entanglements and hygroscopic salts, our FMCHs preserve its polymer‐network architecture without chain scission under cyclic loading, during which the polymer chains slide smoothly against each other with the retained water molecules ensuring its low friction. In contrast, the entangled hydrogel (control sample 1) maintains its birefringence Δ*n* up to 900 cycles; but drastically increases as the cycle number *N* further increases. The increase of the control sample 1′s birefringence is mainly attributed to its dehydration, a common issue faced by most hydrogels.^[^ ^34^ ^]^ We also measure the birefringence of the tough hydrogel (control sample 2), which decreases drastically within a few cycles. This is because the tough hydrogel suffers from fatigue fracture under cyclic loads,^[^ ^18^, ^19^ ^]^ during which the polymer‐network architecture gradually deteriorates due to the depletion of mechanical dissipation, leading to variable color‐changing responses over cycles.

We further perform static relaxation photoelastic characterizations to evaluate our FMCH's rate‐dependent mechanoresponsive color‐changing performance and compare its performance to typical viscoelastic gels. As illustrated in Figure 4d, a step stretch is applied instantaneously to the FMCH and maintained for 1000 s, during which both the force *F* and the birefringence Δ*n* are recorded. As shown in Figure 4e, our FMCH exhibits a slight reduction of force of approximately 5% and stabilizes within seconds, indicating its nearly elastic response. In contrast, viscoelastic gel experiences a force reduction of 40% within the first 40 s, continuing to decrease to 50% over the following 1000s. To further examine the rate‐independent mechanoresponsive color‐changing performance of our FMCH, we vary the loading rate in the static relaxation photoelastic tests. Figure 4f plots the normalized birefringence Δ*n*/Δ*n*_0_ of our FMCH and the viscoelastic gel (control sample 3) at a stretch ratio λ = 3 across different loading rates. Our FMCH demonstrates remarkable stability across different strain rate (Figure S11, Supporting Information), in contrast to the viscoelastic gel, which exhibits increasing birefringence with loading rate. This rate‐independent photoelastic behavior shows our FMCH's potential for tactile applications that require consistent response under dynamic loadings, a significant challenge faced by most polymer‐based photoelastic materials due to their inherent viscoelasticity.

### 2.6 Tactile Sensor toward Vision‐Based Tactile Robots

**Figure 5:**
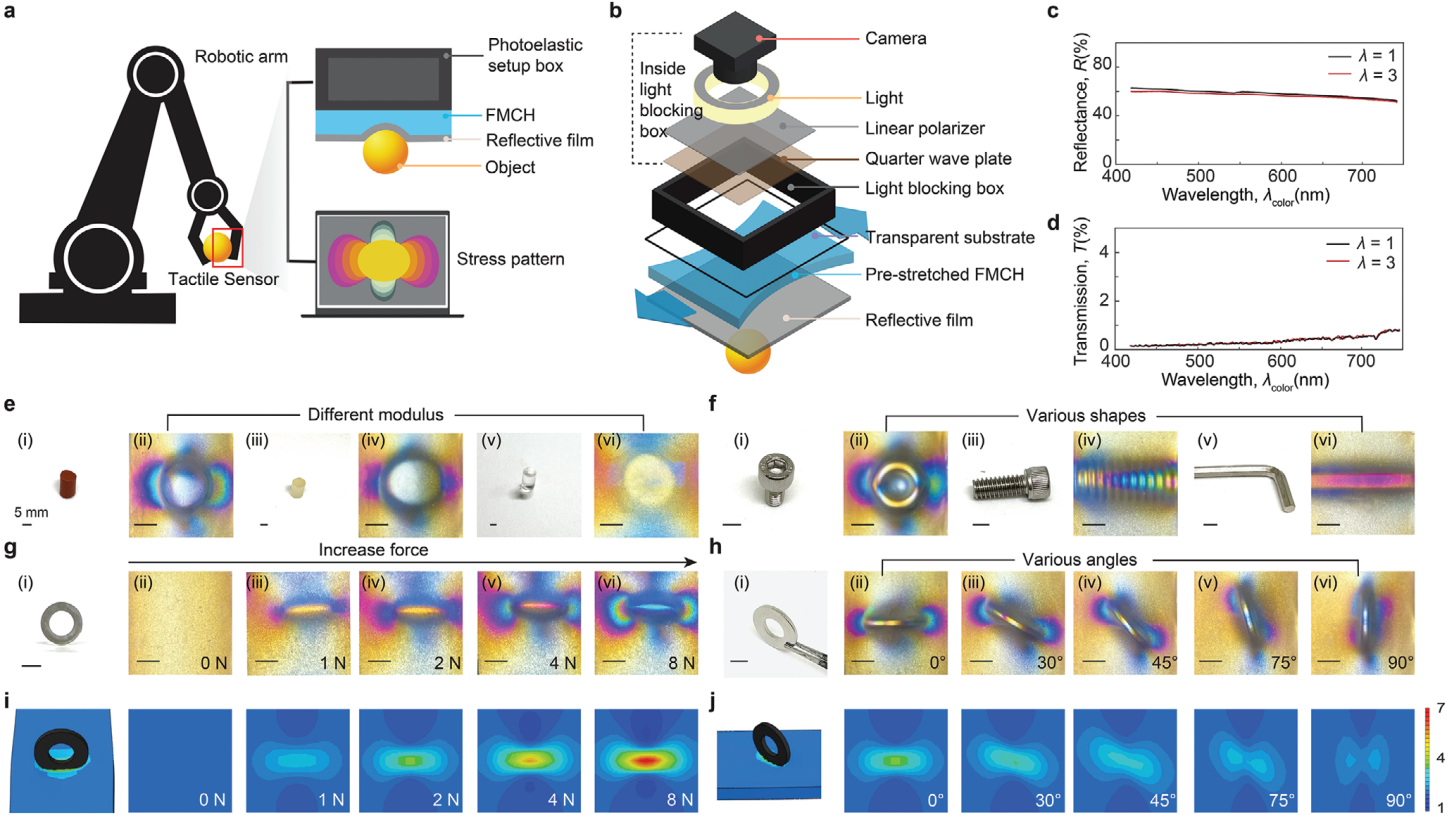
Application of the FMHPs for vision‐based tactile robots. a) Schematic illustration of implementing the FMHP as tactile sensors for vision‐based tactile robots by translating stress distribution into discernible images. b) Schematic illustration of the tactile sensor integrating an imaging system, polarizers, a transparent substrate, pre‐stretched FMHP, and a reflective film. c) Reflectance versus wavelength of light of the soft reflective film at undeformed and stretched state (λ  =  3). d) Transmission versus wavelength of light of the soft reflective film at undeformed and stretched state (λ = 3). e) Different objects with various modulus (i.e., 3 MPa, 68 kPa, 5 kPa). f) Different objects with various shapes (i.e., screw head, thread, hex arm). g) Same object (i.e., flat washer) with increasing forces (0, 1, 2, 4, 8 N). h) Same object (i.e., flat washer) with various angles (0, 30, 45, 75, 90°). i) Stress distributions (difference between principal stress) on FMCH when the flat washer subjected to increasing force (0, 1, 2, 4, 8 N) in simulation, compared to photoelastic images in g). j) Stress distributions (difference between principal stress) on FMCH when the flat washer subjected to increasing force (0, 1, 2, 4, 8 N) in simulation, compared to photoelastic images in h).

We demonstrate one application of this FMCH as a tactile sensor for vision‐based tactile robots, enabling them to discern material stiffness, object shape, spatial location, and applied pressure by translating stress distribution on the contact surface into discernible images. Vision‐based tactile robots,^[^ ^5^, ^35^ ^]^ an emerging class of robotic systems that employ embedded cameras to perceive objects from touch in a visual manner,^[^ ^36^ ^]^ represent a significant advancement in robotic technology for perceiving and interacting with surrounding objects^[^ ^37^ ^]^ (**Figure** **5**a). One key element in vision‐based tactile robots involves the design of color‐changing elastomers as the coupling interface between robots and objects, enabling the translation of tactile sensations into visible color changes. Current methods primarily rely on strain sensing of these elastomers either through photometric stereo or by tracking embedded markers.^[^ ^5^, ^38^ ^]^ While effective for detecting the morphology of rigid objects (modulus higher than that of coupling elastomers) due to modulus mismatch, they are unsuitable for soft objects (modulus lower than that of coupling elastomers), where significant strain occurs directly on the objects rather than the elastomers. Moreover, most coupling elastomers often require frequent calibrations due to material degenerations and environmental factors, particularly under prolonged dynamic stress. In this section, we report the design of an FMCH‐based tactile sensor ensuring consistent and resilient translation of mechanical and physical feedback into discernible color alternations.

Different from the traditional transmission photoelasticimetry setup (Figure S2, Supporting Information), our FMCH‐based tactile sensor employs a reflective photoelasticimetry setup, resulting in a compact design that can be readily integrated into a robotic gripper.^[^ ^39^ ^]^ As schematically illustrated in Figure 5b, the tactile sensor consists of two main systems (Figure S12, Supporting Information): 1) an optical imaging system, which includes a camera, a white light source, a linear polarizer, and a quarter waveplate, all encapsulated in an optical enclosure, and 2) a coupling material system, which includes a rigid transparent substrate, a pre‐stretched FMCH layer, and a stretchable reflective film. The optical enclosure provides an ideal dark environment by preventing external light interference. Furthermore, the pre‐stretch of the FMCH layer enhances the color‐changing sensitivity in response to mechanical deformations. The reflective layer allows the camera and light source to be on the same side, thus enabling a compact design. As the tactile sensor interacts with an object, polarized light first transmits through the pre‐stretched FMCH and then reflects upon reaching the reflective layer, converting the stress distribution on the contact surface into discernible images.

In contrast to the traditional transmission photoelasticimetry setup, the reflectance spectrum of the reflective layer plays an important role in establishing the stress‐birefringence relationship. We perform UV‐vis spectroscopy measurements to evaluate the reflectance and transmission of the reflective film at various stretch levels across visible wavelengths. As shown in Figure 5c, the reflective film maintains a consistent reflectance of around 60% across the visible light spectrum (420 nm to 730 nm) under undeformed and stretched state (λ = 3). This strain‐independent and wavelength‐independent reflectance of the reflective film ensures that the measured photoelastic colors, a mixture of light with different wavelengths, still adhere to the Michel‐Lévy beneficence chart. Additionally, the reflective film possesses high opacity to block external light, which creates an ideal dark environment for the imaging system. As shown in Figure 5d, the transmission of the reflective film at visible wavelengths remains lower than 1% in both its undeformed and stretched states (λ ;= 3). To establish the stress‐birefringence relationship of the tactile sensor, we conduct uniaxial tensile tests on the FMCH, with and without reflective film respectively, recording its corresponding photoelastic images. Furthermore, the reflective film, which is placed at the outermost layer in the tactile sensor, provides a sealed environment for the FMCH hydrogel, reducing the impact of air humidity on its mechanical and optical properties. Unlike the traditional photoelasticimetry method (Figure S2, supporting Information), we position the light source and camera on the same side, with the reflective film placed behind the FMCH. Due to the reflective properties of the film, the polarized light passes through the deformed FMCH twice. As shown in Figure S13 in Supporting Information, the path difference of the FMCH with the reflective film is nearly double compared to the FMCH without the reflective film.

To evaluate the performance of the vision‐based tactile sensor, we conduct indentation tests on objects with varying moduli and different geometries (Figure 5e,f). The tactile sensor exhibits a uniform yellow color without indentation, due to the uniaxial pre‐stretching of the FMCH (Figure 5g‐ii). Firstly, to assess the sensor's performance on different materials, ranging from hard to soft, we apply the same normal loads on three cylinders (i.e., polyurethane, silicone rubber, hydrogel) of identical dimensions but with varying moduli of 3 MPa, 68 kPa, and 5 kPa. When the interacting object is hard, such as the polyurethane cylinder shown in Figure 5e‐i, the contact surface of tactile sensor undergoes large deformation to conform to the object's shape, resulting in a pronounced stress pattern (Figure 5e‐ii). In contrast, when the interacting object has modulus comparable to FMCH, such as the silicone rubber cylinder in Figure 5e‐iii, both the object and the contact surface of sensor undergo deformation, which leads to a subtler stress pattern (Figure 5e‐iv) when compared with polyurethane cylinder. Additionally, when the interacting object is softer than the FMCH, such as the hydrogel cylinder shown in Figure 5e‐v, the sensor's surface experiences even less deformation, but a stress pattern can still be captured by the sensor (Figure 5e‐vi). The distinct stress patterns captured with different material moduli (Figure 5e‐ii,iv,vi) highlight a potential use of the tactile sensor: to establish a correlation between the pattern and modulus through indentation experiments conducted under identical geometry and force with materials of varying moduli. This correlation can then be used to infer the material properties of an object upon contact by analyzing the resulting stress pattern (Figure S14, Supporting Information). Second, to demonstrate the sensor's ability to distinguish different geometries, we apply the same normal load on three different shapes (i.e., screw head, thread, arm hex) with same material (i.e., steel). Figure 5f shows the objects and corresponding captured stress pattern. The overall pattern aligns with the object geometry while the local color corresponds to the stress level. This is especially evident in the case of pressing an inclined thread (Figure 5f‐iii). The patterns of the threads are similar, but due to the inclined angle, each thread experiences a different stress level, leading to variations in color. In contrast, when applying force to an object with a more uniform shape, like a flat surface of hexagonal wrench (Figure 5f‐v), the resulting pattern exhibits a uniform color distribution. The captured image not only reveals the shape of the indented object but also provides information about its three‐dimensional geometry, which is related to the stress pattern.

In addition to evaluating the performance of the indented object with varying geometries and moduli, we further investigate the performance of the tactile sensor on the same object subjected to different forces and at varying positions. Firstly, as shown in Figure 5g, we apply increasing normal force, ranging from 1 to 8 N, on the same object (i.e., flat washer in Figure 5g‐i). As the applied force increases, the contact surface of the tactile sensor experiences larger deformation, resulting in significant changes in birefringence due to the increased stress, and producing a more pronounced stress pattern. Theoretically, the photoelastic stress pattern is correlated with the stress distribution of hydrogel in the deformed state. To validate this relationship, we use Abaqus software to simulate the contact interaction between the FMCH and the flat washer. As shown in Figure 5i, the stress distribution (i.e., difference between principal stress) on the FMCH forms semicircular patterns along the horizontal axis. As the contact force increases, these semicircular patterns extend outward from the center. Due to the uniaxial pre‐stretching of the FMCH, it displays a uniform stress distribution prior to contact with objects. When the FMCH contacts the flat washer, the stress values exceed the initial pre‐stretch value along the horizontal direction, while the stress values fall below the initial pre‐stretch value along the vertical direction. The simulation results closely align with our photoelastic stress patterns: the contact region forms semicircular stress patterns, with more vibrant photoelastic colors along the horizontal direction and darker colors along the vertical direction. Notably, the photoelastic pattern is related not only to the stress level but also to the material's thickness. As shown in Figure S15 (Supporting Information), the central region experiences a significant reduction in thickness, resulting in a less pronounced color change compared to the stress distribution indicated in the simulation results. Additionally, as shown in Figure 5h, we conduct the indentation test on the same object (i.e., flat washer) at different rotating angles, ranging from 0 degrees to 90 degrees, when subjected to an identical force. Due to the uniaxial pre‐stretch of FMCH, the stress distribution varies even interacting with the same object at different positions, which leads to distinct patterns captured by the sensor's camera. This feature allows the tactile sensor to provide accurate positional information about the object, which is essential for robotic manipulation. To enhance the correlation between the simulation results and the photoelastic patterns, we simulate the contact between the hydrogel and flat washer at various rotating angles (Figure 5j). Due to the presence of uniaxial pre‐stretch, the difference in principal stress varies with the rotation position, aligning closely with the experimental results.

By conducting a series of indentation tests with different objects of varying geometries and moduli, as well as with the same object under different forces and positions, we can readily distinguish the object's shape, spatial position, applied pressure, and material stiffness by directly observing the photoelastic stress patterns. Moreover, by comparing these patterns with simulation results, we find that the photoelastic patterns captured by the tactile sensor accurately represent information about stress distribution. This correlation aligns with the actual stress distribution without requiring complex data post‐processing. In the next step, we will design a physics‐informed machine learning algorithm to decode the captured images and infer the material stiffness and geometry of the object, as well as the applied force and spatial position from the stress patterns. In summary, we leverage the high fatigue resistant as well as long‐lasting mechanical and optical properties of FMCH to design a highly compact and simple tactile sensor with cost‐effective components (Cost Analysis in Supporting Information), positioning it as a next‐generation tactile sensor.

## 3 Conclusion

This study presents a fatigue‐resistant mechanoresponsive color‐changing hydrogel (FMCH) featured by its controlled molecular entanglements and adjustable hygroscopic salts. Due to this molecular design, the long polymer chains of FMCH slide against each other and collectively bear force without scission when subjected to mechanical deformation. Additionally, the mechanical properties of hydrogel can be modulated by molecular entanglement, which is negatively related with crosslinker density, and the photoelastic optical property can be tuned by the water content, which is positively related with hygroscopic salts. Moreover, through the monotonic fracture test and cyclic fatigue test, the FMCH exhibits low hysteresis toughening with a fracture toughness is approximately 3000 J m^−2^ and fatigue threshold above 400 J m^−2^. Furthermore, the experiment results, including cyclic dynamic loading, stress relaxation, and various loading speed tests, have demonstrated that FMCH exhibits attributes of long‐term stability and rapid short‐term responsiveness. Boasting these remarkable fatigue resistance and long‐lasting mechanical and optical performance, FMCH emerges as an ideal choice for long‐term dynamic stress sensing. Finally, we present a low‐cost, long‐lasting, vision‐based tactile sensor using FMCH to distinguish object shape, spatial position, applied force, and material stiffness through visualizing photoelastic stress patterns directly. Due to the excellent material properties and simple design, the tactile sensor holds great promise for next‐generation perceptive soft robots.

## Conflict of Interest

J.L., S.L., and W.L. have a pending patent on the design of fatigue‐resistant photoelastic material and vision‐based tactile sensor.

## Author Contributions

J.L., S.L., and W.L. conceived the concept and designed the experiments. J.L. fabricated the sample and performed the experiments. J.L. and S.B. conducted the simulations. J.L. and S.L. wrote the original draft. J.L., S.L., W.L., and Y.S. revised the manuscript.

## Supporting information



Supporting Information

Supplemental Movie 1

## Data Availability

The data that support the findings of this study are available from the corresponding author upon reasonable request.
