# Supplementary material for: Fatigue‐Resistant Mechanoresponsive Color‐Changing Hydrogels for Vision‐Based Tactile Robots
Source: Adv Mater. 2024 Sep 27;37(49):2407925. doi: 10.1002/adma.202407925 (PMC12691901; doi:10.1002/adma.202407925)
Supplement: Supplementary file 1 — Supporting Information [file ADMA-37-2407925-s002.pdf]

ADVANCED MATERIALS

Supporting Information

for *Adv. Mater.*, DOI 10.1002/adma.202407925

Fatigue-Resistant Mechanoresponsive Color-Changing Hydrogels for Vision-Based Tactile Robots

*Jiabin Liu, Wei Li, Yu She, Sean Blanchard and Shaoting Lin**

Supplementary Materials for

Fatigue-resistant Mechanoresponsive Color-changing Hydrogels for Vision-based Tactile Robots

The PDF file includes:

Supplementary Notes

Figs. S1 to S15

Notes for movie S1

References

Other Supplementary Material for this manuscript includes the following:

Movie S1

Supplementary Notes

Materials and Methods

Acrylamide (AAm, Sigma-Aldrich A8887), N,N'-Methylenebisacrylamide (MBAA, Sigma-Aldrich 146072), ammonium persulfate (APS, Sigma-Aldrich A3678), N,N,N',N'-tetramethylethylenediamine (TEMED, Sigma-Aldrich T9281), lithium chloride (LiCl, Sigma-Aldrich L9650), glycerol (Sigma-Aldrich G9012), alginic acid sodium salt (Sigma-Aldrich A2033), and calcium chloride (Sigma-Aldrich C4901) used in this work were purchased from Sigma-Aldrich and used without modification. Hydrogel molds were made of acrylic sheets (8560K191, 8560K171) obtained from McMaster-Carr. The polyurethane rubber rod (80A durometer) for indentation tests was also bought from McMaster-Carr, while the thread was purchased from Grainger. The silicone rubber rod was made using Ecoflex 00-30 (Amazon). The linear polarizing film (XP44-40) and λ/4 retarder film (WP140HE) used in the photoelastic setup and the vision-based tactile sensor were purchased from Edmund Optics. For the homemade photoelasticity test setup, the white LED panel light (4000K light temperature) was bought from eBay, and a Nikon-D800 camera was used to take photos. For the vision-based tactile sensor, the Ecoflex (00-30), mirror spray, USB camera, and black matte tape used in the setup were purchased from Amazon. The LED ring light was purchased from Adafruit.

Synthesis of fatigue-resistant mechanoresponsive Color-changing hydrogel (FMCH)

The pre-gel solution of PAAm-hydrogel was prepared by dissolving 2.5 g of AAm monomer in 2.5 mL of deionized water. A 0.1M APS solution was used as the thermal initiator, 0.23 wt% MBAA as the crosslinker, and TEMED as the crosslinking accelerator. We added 80 μL of MBAA, 40 μL of APS, and 10 μL of TEMED to 5 g of the pre-gel PAAm solution. To synthesize hydrogels with varying crosslinker densities, the volume of MBAA was adjusted from 40 μL to 400 μL. The mixture was vortexed for 2 minutes, then poured into an acrylic mold sealed with an acrylic cover. The mold and cover were cut using a laser cutter (Epilog Fusion Laser Cutter). The precursors were then placed on a hotplate at 50°C for 2 hours for thermal curing. To avoid the long swelling time required in high-concentration LiCl solutions, the synthesized hydrogels were first immersed in deionized water until they reached equilibrium. Following this swelling process, the hydrogels were then soaked in LiCl solutions of varying concentrations, ranging from 2M to 16M, and left overnight to ensure complete absorption of the salt particles. The hydrogels containing LiCl solutions were then exposed to ambient environment to reach an equilibrium state.

Synthesis of Common Hydrogels

Control sample 1 was produced from the same precursor and underwent the same curing process as the FMCH. The synthesized hydrogel was soaked in deionized water until it reached equilibrium, without being immersed in the LiCl solution. The fully swollen hydrogel was then dried at room temperature for several hours before testing.

Control sample 2 was prepared by mixing 20 mL of deionized water, 2.4 g of AAm, and 0.4 g of alginate. To this, 2 mL of 0.23 wt% MBAA, 500 μL of 0.1M APS, and 20 μL of TEMED were added to the 20 mL pre-gel solution. The mixture was vortexed for 30 seconds, then poured into a mold and sealed with a cover. The precursors were placed on a hotplate at 50°C for 0.5 hours for thermal curing. After curing, the gel was soaked in a 0.1M CaCl2 solution for 1 hour.

Control sample 3, used in the relaxation test, was made of acrylamide, with glycerol serving as the hygroscopic solution. A pre-gel solution was prepared by mixing 6 mL of glycerol, 10 mL of deionized water, and 5 g of AAm monomer. To this, 1 mL of 0.23 wt% MBAA, 150 μL of 0.1M

APS, and 20 µL of TEMED were added to 10 mL of the pre-gel solution. The mixture was vortexed for 1 minute, then poured into a mold and sealed with a cover. The precursors were placed on a hotplate at 50°C for 0.5 hours until curing. After curing, the hydrogel was dried at room temperature overnight until its weight reached equilibrium.

Mechanical Tests for Hydrogels

For uniaxial mechanical test, the hydrogels were made as a dog-bone shape, and the acrylic mold size is 10 mm × 30 mm × 3 mm. For the fracture and fatigue mechanical test, the hydrogels were made as a rectangular shape with a size of 10 mm × 60 mm × 3 mm. The grippers of hydrogel at top and bottom were made by acrylic board. The grippers were glued with hydrogel samples using Krazy glue and the mechanical tests were performed by a universal testing machine (CellScale Testing Machine).

To get the stretch-stress and stress-birefringence curve of hydrogels, the hydrogels was subjected to uniaxial loading monotonically till fracture and the photoelastic images of samples were recorded by camera every second. To get the fracture toughness of hydrogel, the hydrogel samples were made as a rectangular shape, and the unnotched sample was stretched monotonically till fracture with the force and displacement recorded. Then, a notched sample with same dimensions was monotonically loaded till critical stretch ratio λ_c. The fracture energy was calculated using equation $G = H\int_1^{\lambda c} S d\lambda$, where H is the height of sample, S and λ is the normal stress and stretch ratio in the pure shear test with unnotched sample.

For the fatigue test, a rectangular sample (10 mm × 60 mm × 3 mm) was cut with an initial crack length 1 mm along the middle line along the horizontal direction by a blade. The sample was applied by cyclic loading with various stretch ratios, ranging from 0.5 to 3. The crack length of the sample was measured during every 100 cycles with small stretch ratio and 10 cycles with large stretch ratio. To get the value of crack extension per cycle (dc/dN), the total crack extension length was divided by the cycle number, with an assumption that the extension is linearly increase with cycle number. To test the photoelastic color under long-term cyclic loading, uniaxial testing was applied on three hydrogels (FMCH, common hydrogel 1 and 2) and the photoelastic color at the same location was recorded at the same stretch ratios. To get force data with time in the static relaxation experiments, the two hydrogel samples (FMCH, common hydrogel 3) were stretched to a certain ratio with various loading speeds. The color during stretching with various speed, ranging from 0.5 mm/s to 10 mm/s was recorded by camera every second. After loading, the samples were hold at certain stretch ratio for 1000 seconds. The force and corresponding photoelastic color were recorded.

The Degree of Entanglement

Through comparison with regular PAAm hydrogel, the degree of entanglements can be estimated in the FMCH material. Firstly, we assume all the crosslinkers in the precursor solution are incorporated into the polymer. Each crosslinker is an end of four chains and each chain has two ends. For the hydrogel with ϕ_c = 3.3×10^{-5}, which ϕ_c is the crosslinker to monomer molar ratio. The average number of monomers per chain is about 1.4×10^{4} monomers. The modulus of the fully swollen hydrogel is about 5 kPa. From literature, the modulus is the same with the low-entangled PAAm hydrogel with crosslinker ϕ_c = 9.2×10^{-3}, which has an average number of monomers per chain about 55. Therefore, each chain of FMCH has entanglements equivalent to 200 crosslinkers when compared with regular low-entangled PAAm hydrogel.

The Circular Polariscope

The circular polariscope is a photoelastic instrument[1] used to analyze birefringence properties. Compared to a linear polariscope, which is composed solely of two crossed linear polarizers, a circular polariscope allows simultaneous observation of isoclinic patterns (contours of equal principal stress direction) and isochromatic patterns (contours of equal principal stress difference). This capability is achieved because the circular polarizers eliminate the isoclinic patterns, thereby providing a clearer and more comprehensive view of the stress distribution within the material. The set up of circular polariscope[2] is shown in **Figure S2**. It is composed of a light source, two linear polarizers, two quarter-wave plates, a universal testing machine with photoelastic samples, and a camera. The input light source is an LED panel with a color temperature of 4000K. The first linear polarizer and first quarter-wave plate before the sample are called the polarizer, while the second set is called the analyzer. The two linear polarizers are installed orthogonally to each other to create the darkest possible observed light field. The first quarter-wave plate is installed at a 45-degree angle, and the second quarter-wave plate is orthogonal to the first to darken the observed light field again. The natural white light from the LED panel is considered light with arbitrary transverse vibrations. The linear polarizer allows light with a specific direction to pass through, and the quarter-wave plate introduces a phase difference of 90 degrees between the orthogonal components of light. After passing through the polarizers, the incident light becomes circularly polarized light with equal amplitude in orthogonal directions. When the circular polarized light meets with the sample under mechanical force, a retardation δ will be introduced into the light. δ is dependent on the difference between principal stress and direction. After passing through the analyzers, the light intensity will follow:

$$I = I_0\, sin^2(\pi\delta/\lambda) \tag{S1}$$

$$\delta = \Delta nd \tag{S2}$$

where I_0 is the input intensity, Δn is the birefringence, d is the thickness of sample under mechanical force, λ is the wavelength of light. The intensity of light with different wavelengths depends on its specific wavelength. When the light of various wavelengths mixes, an interference color is often observed. The theoretical intensity of R, G, B channel after calibration[3] is shown in **Figure S7b**. We used the experimental color to compare with the theoretical RGB and Michel-Levy Birefringence Chart[4] (**Figure S7**). The unknown retardation was found by minimizing an error function:

$$e_i = \sqrt{(R - R_i)^2 + (G - G_i)^2 + (B - B_i)^2} \tag{S3}$$

where R, G, B are the three channels of theoretical color, R_i, G_i, B_i is three channels of experimental color. After determining the retardation, the birefringence value can be obtained by dividing the retardation by the thickness of the sample after it has been subjected to mechanical force.

Nonlinear Photoelastic Theory

In the case of large deformation of hydrogel, both the mechanical and optical anisotropy changed with the network geometry. Arruda used an eight-chain network theory[5], which considers the extensibility of polymer chains, to describe the nonlinear relationship between stress and birefringence. In their theory, they used the non-Gaussian chain assumption, which considered the deformed chain length will approach its contour length, and then used the eight-chain theory to describe the deformation of polymer network. In an optical medium, the refractive index n can be quantified by the polarizability P using Lorentz-Lorenz equation:

$$\frac{n_i^2-1}{n_i^2+2} = \frac{4\pi}{3}P_i \tag{S4}$$

where n_i is the refractive index of principal direction, P_i is the polarizability. The birefringence Δn, which is the difference between two principal refractive index $n_1 - n_2$, can be expressed by the difference of principal polarizability[6]:

$$\Delta n = \frac{2\pi}{9}\frac{(n_0^2+2)^2}{n_0}(P_1 - P_2) \tag{S5}$$

where n_0 is the average refractive index, given by $\frac{n_1+2n_2}{3}$ under simple tension[6b], P_1 and P_2 are polarizability of polymer network along two perpendicular principal directions. The eight-chain model is to conceptualize bulk material as a network of eight interconnected chains in a representative cubic. Each of these units has eight chains, all connected at the center of a cube. The unit polarizability is derived by non-Gaussian chain theory with single monomer polarizability α. The network polarizability P_i is the summation of the polarizability of each unit p_i. The network polarizability difference[7] can be calculated as:

$$P_1 - P_2 = nN(\alpha_1 - \alpha_2)\left(\frac{\lambda_1^2-\lambda_2^2}{3\Lambda^2}\right)\left(1 - \frac{3\Lambda/\sqrt{n}}{L^{-1}(\Lambda/\sqrt{n})}\right) \tag{S6}$$

where n is the chain length, N is the number of chains per volume, α_1, α_2 are the monomer polarizability along principal directions, $\Lambda = \sqrt{(\lambda_1^2 + \lambda_2^2 + \lambda_3^2)/3}$, and $L^{-1}(x)$ is the inverse Langevin equation. The birefringence is calculated using Eq. (S5) and Eq. (S6),

$$\Delta n = \frac{5}{3}CN\left(\frac{L^{-1}(\Lambda/\sqrt{n})n - 3\Lambda\sqrt{n}}{\Lambda^2 L^{-1}(\Lambda/\sqrt{n})}\right)(\lambda_1^2 - \lambda_2^2) \tag{S7}$$

C is the stress-optical coefficient of materials. Based on the incompressible eight-chain model, the stretch ratio has relationship with true stress:

$$\sigma_1 - \sigma_2 = \frac{NkT}{3\Lambda}\sqrt{n}L^{-1}(\Lambda/\sqrt{n})(\lambda_1^2 - \lambda_2^2) \tag{S8}$$

Where k is the Boltzmann constant, T is the temperature. Depend on Eq. (S7) and Eq. (S8), the relationship between birefringence and stress difference can be expressed:

$$\frac{n_1-n_2}{\sigma_1-\sigma_2} = 5C\frac{1}{kT}\frac{\sqrt{n}L^{-1}(\Lambda/\sqrt{n})-3\Lambda}{\Lambda L^{-1}(\Lambda/\sqrt{n})^2} \tag{S9}$$

We use Eq. (S9) to fit the experimental results to get the stress-optical coefficient C.

Synthesis of reflective film and UV-Vis

To synthesize the reflective film for the tactile sensor, we mixed 2 mL of silver liquid from the mirror spray with 5 mL of Ecoflex 00-30A and 5 mL of Ecoflex 00-30B. The mixture was vortexed for 2 minutes. The precursors were then poured into a petri dish, which was slowly rotated horizontally to form a thin layer of the viscous liquid. The precursor was left at room temperature to cure for approximately two hours.

The UV-Vis measurement was conducted using a Lambda 900 (Perkin Elmer). First, a test was conducted using a highly reflective standard material to calibrate the reflectance. Then, the reflective film, measuring 10 mm in length and 5 mm in width, underwent a UV-Vis test to measure its reflectance and transmission in the visible range, from 400 nm to 780 nm. After testing the undeformed reflective film, we stretched the film along its longer side to lengths of 20 mm, 30 mm, and 40 mm, respectively. The stretched film was placed on a piece of transparent glass and clamped from two sides. Subsequently, we conducted the same reflectance test on the stretched film within the same visible light range.

Digital image correlation (DIC) for crack propagation experiments

Digital image correlation is a non-contact optical technique that allows full-field strain measurement on a surface under deformation[8]. We prepared a fatigue-resistant photoelastic

sample, which is 65mm in length, 12mm in width, and 4mm in thickness, for DIC measurement. We used a sharp blade to make a 25mm long crack in the middle of the sample along its length. Afterwards, we used black paint to apply the speckle patterns on the surface of the sample. Following the spray, the sample was rested for an hour to make sure the speckle pattern adhesive on the surface. To improve the quality of images, a white light panel was set up behind the sample, and a camera was used to capture the deformation process every second. Afterwards, all images are transformed to grey images. To track the surface displacements of deforming materials, a mathematically well-defined correlated function is applied to match digitalized images before deformation and after deformation[9].

Finite Element Analysis for Fracture Test

The finite-element calculations for pure shear fracture test are performed with ABAQUS/Explicit. We implemented the cohesive-zone model and nonlinear deformation model into a two-dimensional finite element model to simulate the pure shear crack propagation of soft materials. The geometry of the soft material is 65mm in length, 12mm in width and 4mm in thickness. The initial crack length was 25mm. The pure elastic property of soft material was fitted by the one term Ogden hyperplastic model[10]. The strain energy density is expressed as

$$W = 2\mu/\alpha_1^2(\lambda_1^{\alpha_1} + \lambda_2^{\alpha_1} + \lambda_2^{\alpha_1} - 3) \quad \text{(S10)}$$

Where W is the strain energy density, $\lambda_1, \lambda_2, \lambda_3$ are three principal stretch ratios, μ is shear modulus, and α_1 is material constant. The soft material is modeled using plane-stress 4-node linear elements with reduced integration. The cohesive model for describing the failure of material is implemented in ABAQUS using cohesive elements. The hydrogel strip is loaded along vertical direction with a constant velocity. The curve of force versus stretch ratio and strain field of simulation were shown in **Figure. S9**.

Finite Element Analysis for Contact between FMCH and object

The finite-element calculations for the contact between the soft material and the contact object were performed using ABAQUS/Static. The contact models consist of two parts: a nonlinear deformation model implemented into a three-dimensional dog-bone-shaped FMCH, and a linear elastic model applied to a flat washer. The simulation comprises two steps. In the first step, the dog-bone-shaped FMCH was uniaxially stretched with a stretch ratio of 1.5. In the second step, the rigid flat washer was moved at a constant speed to gradually contact the pre-stretched hydrogel. The distribution of principal stress difference is shown in **Figure 5** and the displacement field of the FMCH is shown in **Figure S15**.

Cost analysis for vision-based tactile sensor

The vision-based tactile sensor was designed with low cost in mind. Due to the wide variation in digital camera prices depending on their quality, we estimated the cost of other components of the tactile sensor. The cost of each part is summarized below using prices of retail price:

- The cost of the fatigue-resistant mechanoresponsive Color-changing hydrogel (FMCH) in the tactile sensor is approximately \$1.5, including all the chemicals used in material synthesis.
- The reflective film costs about \$0.5, covering the expenses for Ecoflex 00-30 and silver paint.
- The acrylic boards, which include the transparent substrate and the light blocking box, are priced around \$3.
- The linear and quarter-wave polarizers used in the tactile sensor amount to about \$3.

- The high-power white LED chip is approximately $1.

Therefore, the total cost of the tactile sensor setup except camera, is around $9. The cost analysis is based on the retail price and the wholesale price will be even cheaper.

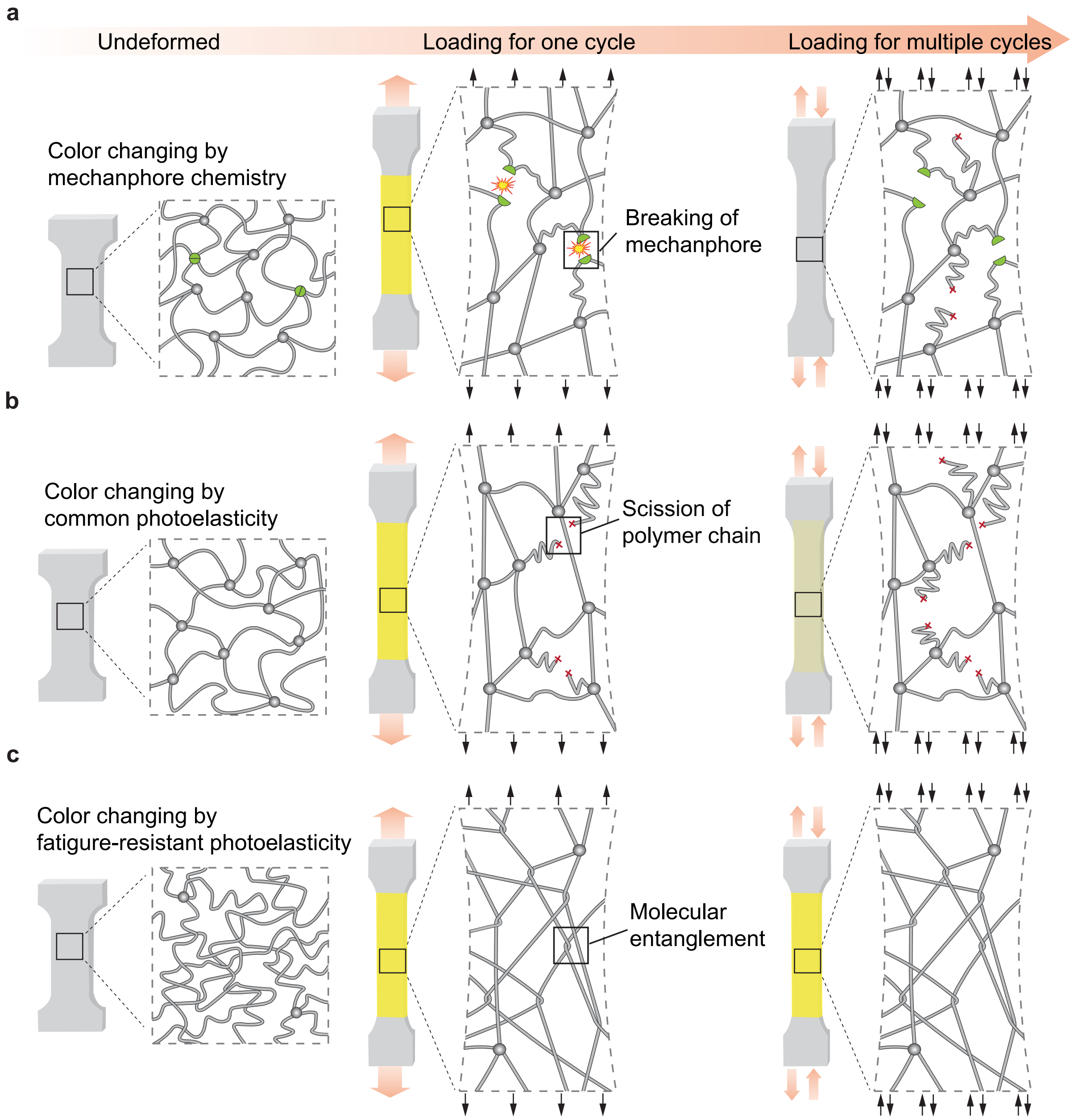

Figure S1. Molecular design of three color-changing materials subjected to single and multiple cycle loadings. a. Polymers containing mechanophores exhibit color changes during the initial loading due to the breaking of mechanophores. However, the color fades after multiple loading cycles. **b.** Common hydrogels exhibit a photoelastic color due to chain alignment during the initial loading. However, this color fades as the polymer chains undergo scission, disrupting their structural alignment. **c.** The fatigue-resistant material FMCH preserves a constant photoelastic color during multiple cyclic loadings, attributed to its design of controlled molecular entanglements and the incorporation of hygroscopic salts.

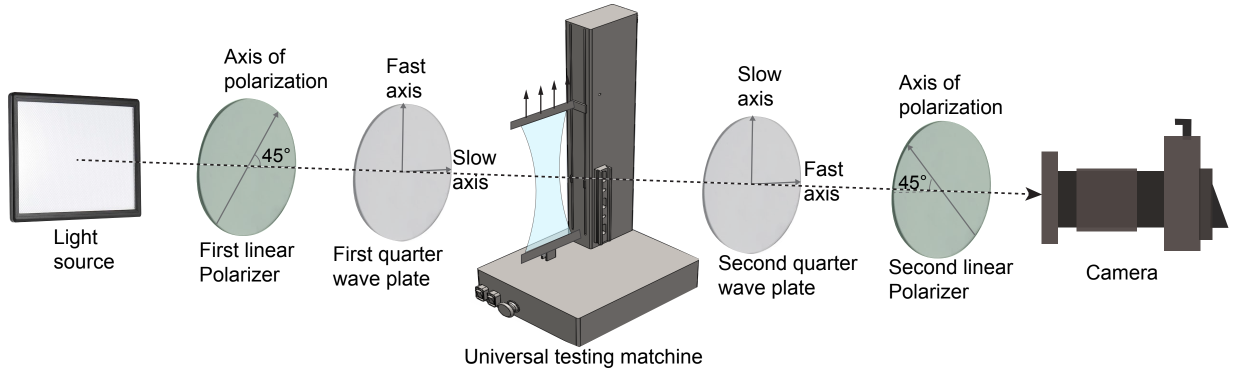

Figure S2. Homemade photoelasticimetry setup. The setup contains a white light source, a camera, two linear polarizers, two quarter-wave plates, a universal testing machine, and a testing hydrogel.

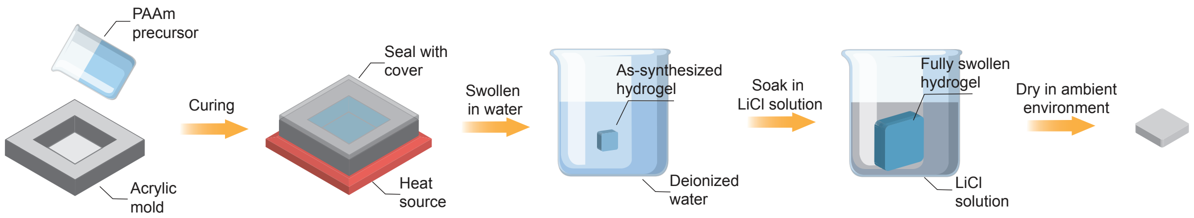

Figure S3. The process of fabricating FMCH. The pre-gel solution is poured into an acrylic mold and is sealed with a cover. The mold is placed on a hotplate at 50°C for about 2 hours to cure. Then, the as-synthesized gel is immersed in deionized water until it is fully swollen. The swollen gel is soaked into a LiCl solution until the salt particles have diffused evenly into the gel. Finally, we dry the gel in an ambient environment until it reaches its weight equilibrium.

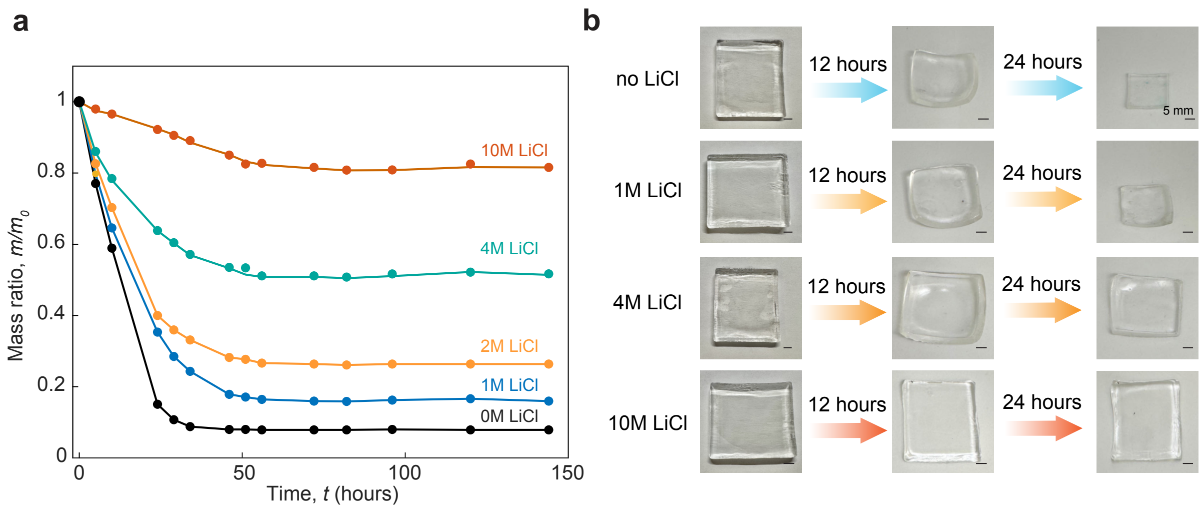

Figure S4. Characterization of water retention in hydrogels. a. The curves of mass ratio versus time for hydrogel samples with different amounts of LiCl exposed in an open environment with 50% humidity. **b.** Images of the hydrogel samples with different amounts hygroscopic salt exposed in an open environment for 12 hours and 24 hours.

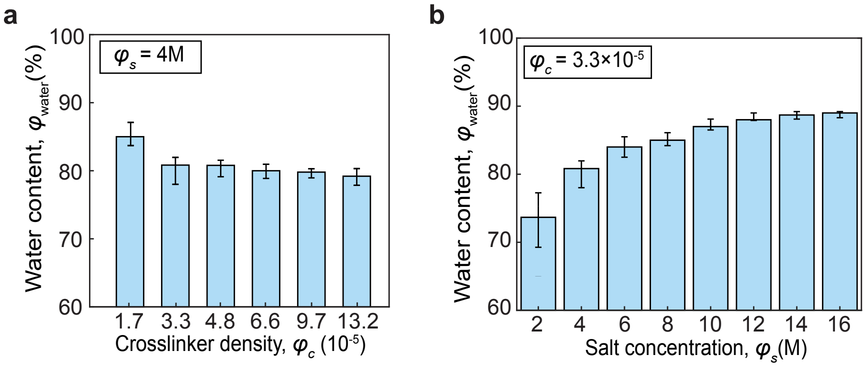

Figure S5. Retained water content. a. Water content of hydrogel φ_{water} versus crosslinker density φ_c with salt concentration $\varphi_s = 4$ M. **b.** Water content φ_{water} versus salt concentration φ_s range from 2 M to 16 M with crosslinker density $\varphi_c = 3.3 \times 10^{-5}$. The humidity in the ambient environment is 50%.

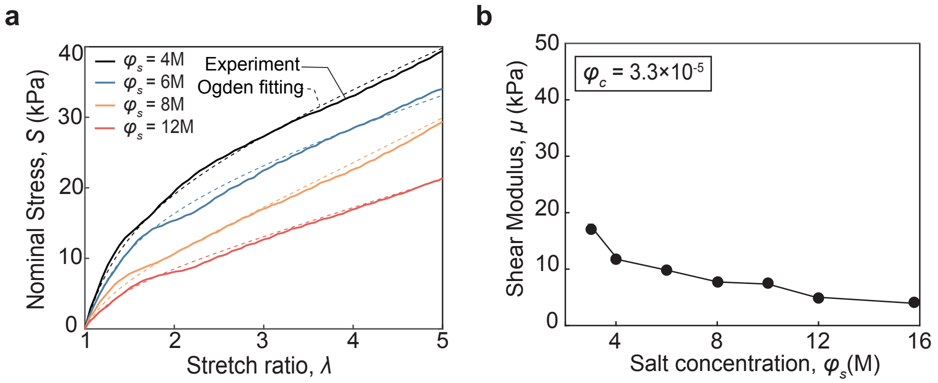

Figure S6. Mechanical characterization. a. The curves of nominal stress versus uniaxial stretch ratio of hydrogel with various salt concentrations (4 M, 6 M, 8 M, 12 M). **b.** Shear modulus slightly decreases with salt concentration increases.

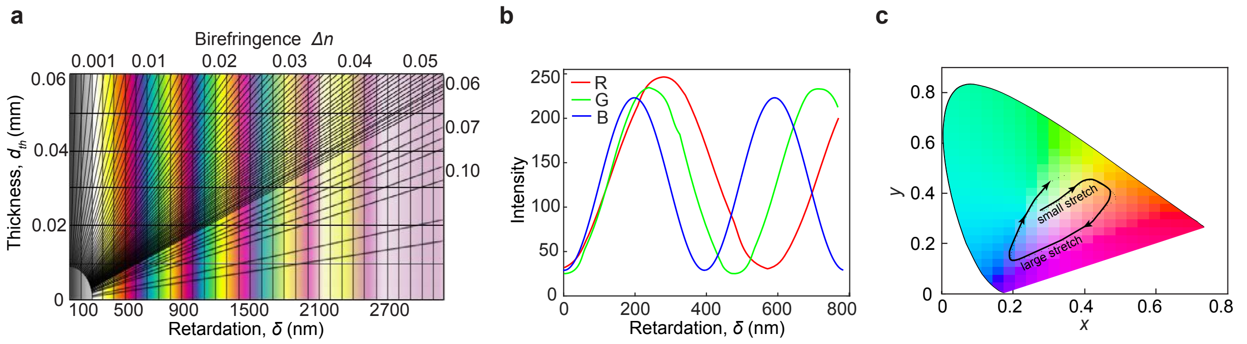

Figure S7. Theoretical and experimental photoelastic colors. a. Michel-Levy Birefringence Chart. **b.** Theoretical value of R, G, B channels versus retardation. **c.** Experimental photoelastic color trajectory marked in CIE 1931 color space. x and y are the chromaticity coordinates.

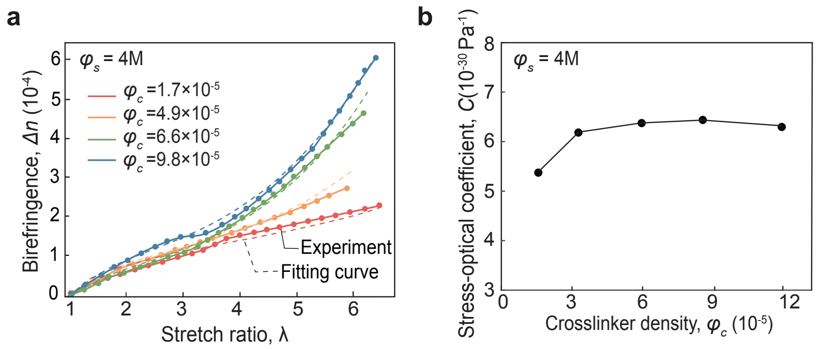

Figure S8. Photoelastic characterization. a. The curves of stretch ratio versus birefringence with various crosslinker density φ_c. **b.** Stress-optical coefficient C slightly increases as the crosslinker density φ_c increases.

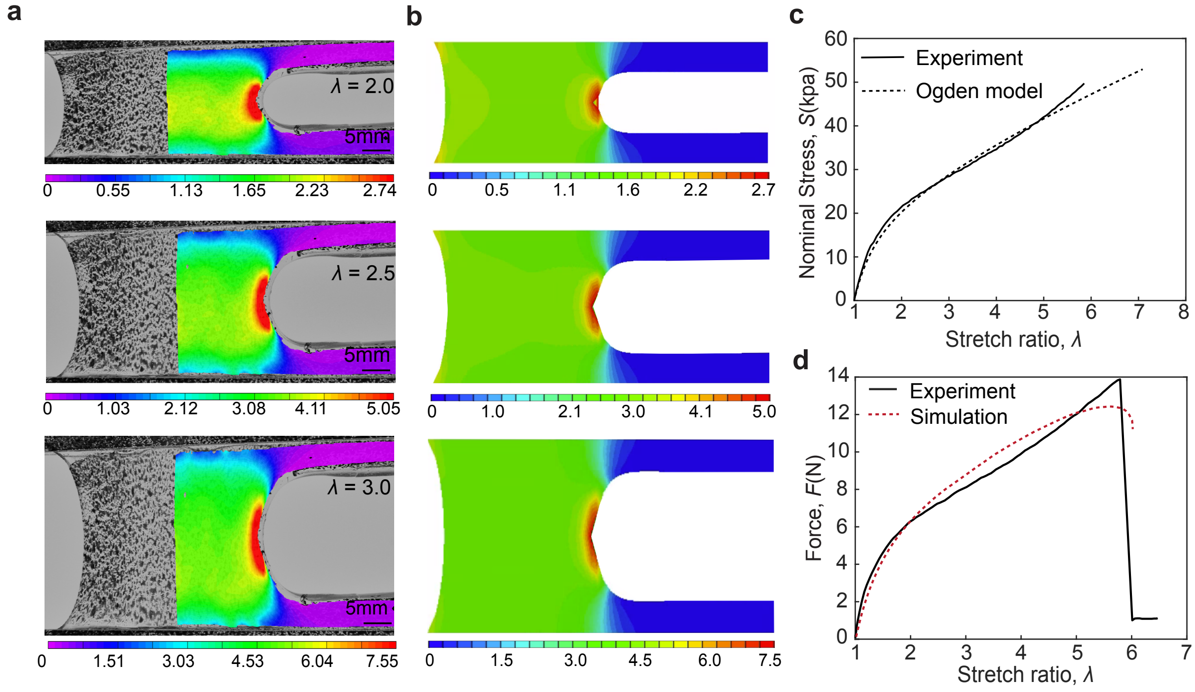

Figure S9. DIC and simulation results. a. Strain field of DIC test under different stretch levels. **b.** Strain field of Abaqus simulation under different stretch levels. **c.** The curve of nominal stress versus stretch ratio of unnotched sample under pure shear tensile test. **d.** The curve of force versus stretch ratio of a notched sample from experiment and simulation.

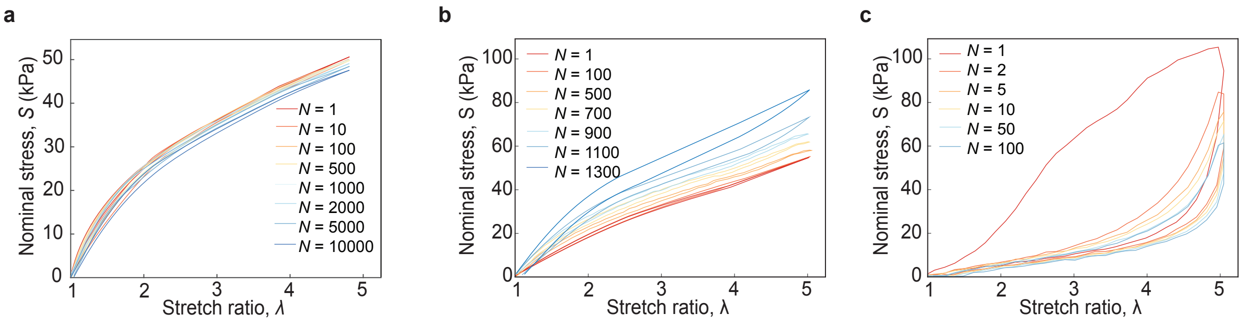

Figure S10. The curves of stretch ratio *λ* versus nominal stress *S* under dynamic cyclic mechanical tensile test. a. Fatigue-resistant mechanoresponsive Color-changing hydrogel (FMCH). **b.** Control sample 1. **c.** Control sample 2.

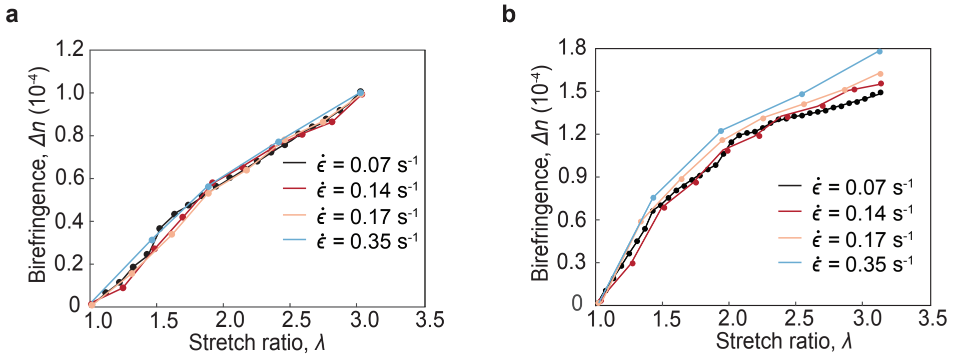

Figure S11. The curves of birefringence versus stretch ratio under various strain rates. a. Fatigue-resistant mechanoresponsive Color-changing hydrogel (FMCH). **b.** Control sample 3.

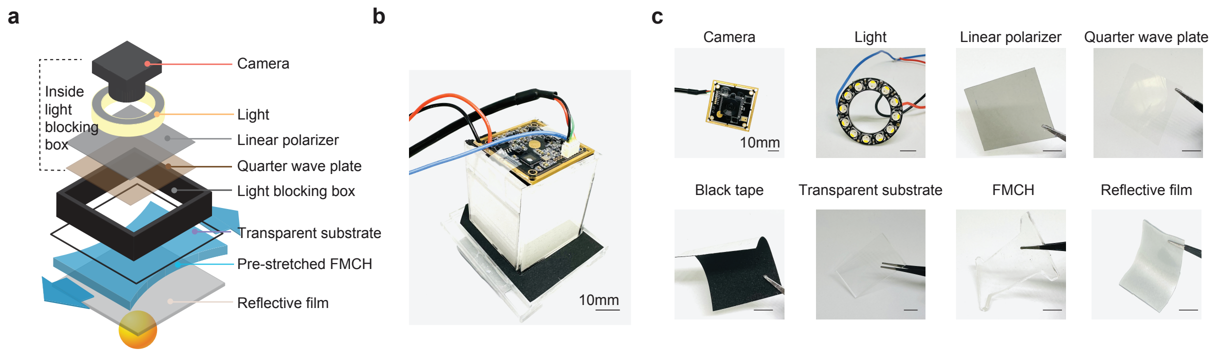

Figure S12. Vision-based tactile sensor setup and images. a. Schematic of the design of tactile sensor. **b.** Image of assembled vision-based tactile sensor. **c.** Images of components, including camera, light, linear polarizer, quarter wave plate, black tape, transparent substrate, FMCH, and reflective film.

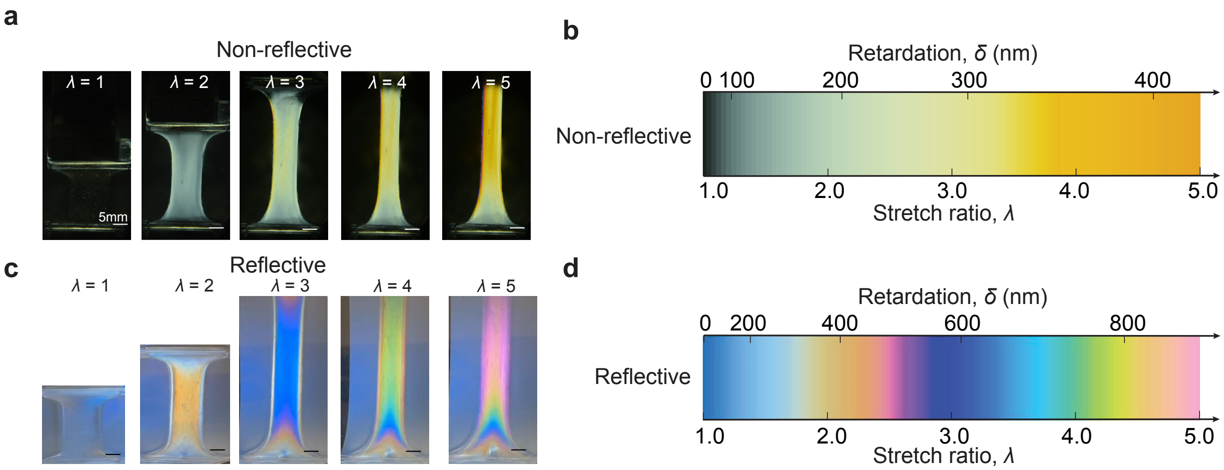

Figure S13. Experimental photoelastic color with and without reflective film. (a) Images of the sample under uniaxial tension, without a reflective film, showing stretch ratios from 1 to 5, captured under a circular polariscope. (**b)** Experimental color and corresponding retardation δ of FMCH without reflective film under various stretch ratios. **(c)** Images of the sample under uniaxial tension, with reflective film, showing stretch ratios from 1 to 5. **d.** Experimental color and corresponding retardation δ of FMCH with reflective film under various stretch ratios. The retardation with the reflective film is approximately twice the value of the retardation without the reflective film.

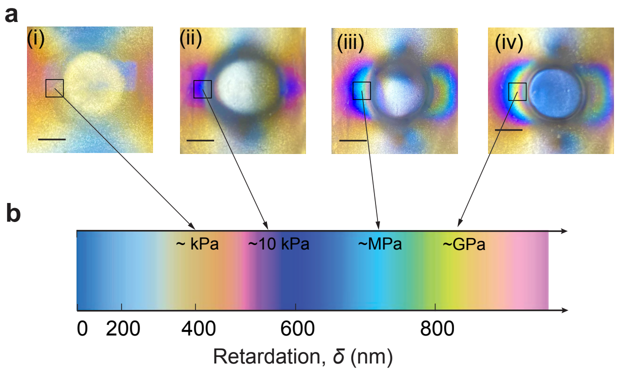

Figure S14. Captured patterns and its corresponding modulus. a. Captured patterns with cylinders of various modulus. **b.** The corresponding modulus range at the experiment color span.

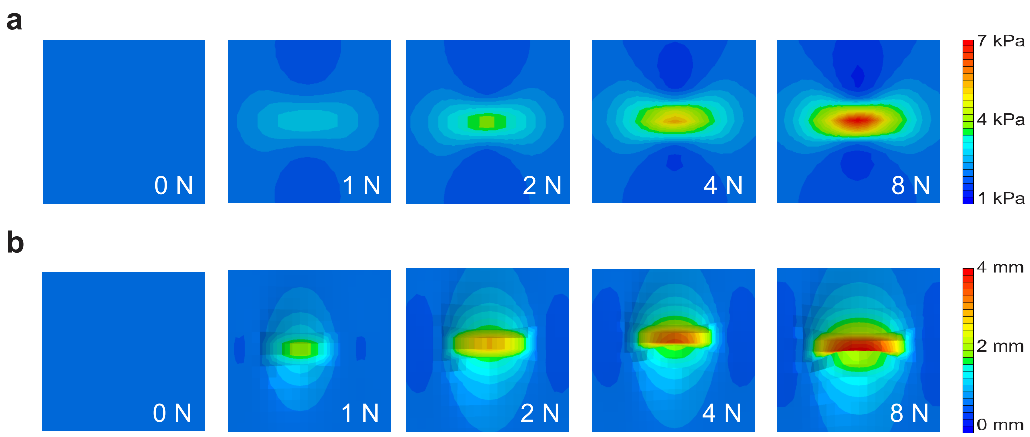

Figure S15. Simulation results of a flat washer in contact with FMCH subjected to various forces. a. Stress distributions (difference between principal stress) on FMCH when the flat washer subjected to increasing force (0, 1, 2, 4, 8 N) in simulation. **b.** Displacement field on FMCH when the flat washer subjected to increasing force (0, 1, 2, 4, 8N) in simulation.

Notes for movie S1

Movie S1. Color-changing performance of FMCH under uniaxial tensile loading.
This video shows that FMCH exhibits color change when subjected to uniaxial tensile loading.

Reference

[1] H. Aben, C. Guillemet, *Photoelasticity of glass*, Springer Science & Business Media, **2012**.
[2] W. Li, Y. Meng, B. K. Primkulov, R. Juanes, *Physical Review Applied* **2021**, 16, 024043.
[3] A. Ajovalasit, G. Petrucci, M. Scafidi, *Optics and Lasers in Engineering* **2015**, 68, 58.
[4] M. W. D. Robert Hoffman.
[5] E. M. Arruda, M. C. Boyce, *Journal of the Mechanics and Physics of Solids* **1993**, 41, 389.
[6] a)D. Sun, T. Lu, T. Wang, *Soft Matter* **2021**, 17, 4998; b)L. G. Treloar, **1975**.
[7] E. M. Arruda, P. A. Przybylo, *Polymer Engineering & Science* **1995**, 35, 395.
[8] W. Peters, W. Ranson, *Optical engineering* **1982**, 21, 427.
[9] H. Bruck, S. McNeill, M. A. Sutton, W. Peters, *Experimental mechanics* **1989**, 29, 261.
[10] R. W. Ogden, *Non-linear elastic deformations*, Courier Corporation, **1997**.
